# Microbiota in sports

**DOI:** 10.1007/s00203-022-03111-5

**Published:** 2022-07-14

**Authors:** Katarzyna Mańkowska, Małgorzata Marchelek-Myśliwiec, Piotr Kochan, Danuta Kosik-Bogacka, Tomasz Konopka, Bartłomiej Grygorcewicz, Paulina Roszkowska, Elżbieta Cecerska-Heryć, Aldona Siennicka, Justyna Konopka, Barbara Dołęgowska

**Affiliations:** 1 Department of Laboratory Medicine, Chair of Microbiology, Immunological Diagnostics and Laboratory Medicine, Pomeranian Medical University, Al. Powstańców Wlkp 72, 70-110, Szczecin, Poland; 2 Clinic of Nephrology, Transplantology and Internal Diseases, Pomeranian Medical University, Szczecin, Poland; 3 Department of Bacteriology, Microbial Ecology and Parasitology, Chair of Microbiology, Jagiellonian University Medical College, Cracow, Poland; 4 Independent of Pharmaceutical Botany, Department of Medical Biology and Parasitology, Pomeranian Medical University, Szczecin, Poland; 5 Department of Orthopedics, Traumatology and Oncology of the Musculoskeletal System, Pomeranian Medical University, Szczecin, Poland; 6 Department of Immunological Diagnostics, Chair of Microbiology, Immunological Diagnostics and Laboratory Medicine, Pomeranian Medical University, Szczecin, Poland; 7 Department of Laboratory Diagnostics, Pomeranian Medical University, Szczecin, Poland; 8 Department of Orthodontics, Pomeranian Medical University, Szczecin, Poland

**Keywords:** Microbiota, Sport, Diet, Physical activity, Sport disciplines, Probiotics

## Abstract

The influence of microbiota on the human body is currently the subject of many studies. The composition of bacteria colonizing the gastrointestinal tract varies depending on genetic make-up, lifestyle, use of antibiotics or the presence of diseases. The diet is also important in the species diversity of the microbiota. This study is an analysis of the relationships between physical activity, diet, and the microbiota of the gastrointestinal tract in athletes. This review shows the differences in the microbial composition in various sports disciplines, the influence of probiotics on the microbiome, the consequence of which may be achieved even better sports results. Physical activity increases the number of bacteria, mainly of the *Clostridiales* order and the genus: *Lactobacillus*, *Prevotella*,* Bacteroides*, and *Veillonella*, and their number varies depending on the sports discipline. These bacteria are present in athletes in sports that require a high VO_2_ max. The players’ diet also influences the composition of the microbiota. A diet rich in dietary fiber increases the amount of *Lactobacillus* or *Bifidobacterium* bacteria, probiotic microorganisms, which indicates the need to supplement the diet with probiotic preparations. It is impossible to suggest an unambiguous answer to how the microbiota of the gastrointestinal tract changes in athletes and requires further analyzes.

## Introduction

In 2007, the Human Microbiome Project (HMP) was initiated, which aimed to determine the microbiota colonizing the human body. For this purpose, samples were collected from five ontocenoses: oral cavity, skin, nasal cavity, gastrointestinal tract and urogenital tract of 250 healthy adult volunteers. The samples were subjected to comparative analysis based on 16S rRNA sequences, and then, databases were created (Olszewska and Jagusztyn- Krynicka 2012). It turned out that the human body is colonized by over 100 trillion symbiotic bacteria weighing more than 2 kg of body weight, the species composition of which may vary depending on the state of health (Pane et al. 2018).

The main role in colonization is assigned to the microbiota of the gastrointestinal tract. It mainly consists of species belonging to five phyla: *Firmicutes, Bacteroides, Actinobacteria, Proteobacteria* and *Verrucomicrobia.* In addition, there is also a large share of bacteria of the genus *Cyanobacteria, Spirochaetes* and anaerobic microorganisms of the genera: *Eubacterium, Bifidobacterium, Clostridium, Peptostreptococcus* and *Ruminococcus* (Lozupone et al. 2012; Sanchez et al. 2017). The composition of the microbiota is influenced by many factors, such as genetics, lifestyle, antibiotic therapy, diet and diseases.

Symbiotic bacteria begin to colonize the host organism shortly after birth. This simple microbial community gradually develops as the host grows. The host-microorganism system is most often an interaction based on mutual benefit. The host organism has favorable conditions for the multiplication of bacteria, while the influence of microbiota on the human body is even greater. First of all, human microbiota protect the human body against pathogens, taking up space for their entry and existence. It is a physiological barrier for pathogenic bacteria, while often producing substances with antibacterial properties (Mauro et al. 2013).

The role of the gastrointestinal microbiota is best understood. Its bacteria break down nutrients, provide the body with essential nutritional substances, stimulate the development of humoral and cellular immune responses, modulate metabolism, and supply the body with specific products of bacterial metabolism. Additionally, a clear correlation was found between the presence and composition of the gastrointestinal microbiota and metabolic diseases such as obesity, inflammatory bowel disease, metabolic syndrome, cirrhosis and liver cancer (Sanchez et al. 2017).

In addition to the factors indicated above, the microbiota composition may also be influenced by physical activity or diet. Therefore, the study aims to analyze the correlation between the composition of the microbiota and physical activity in athletes, which may improve their sports performance.

## Microbiota and physical activity

The latest literature reports indicate a close relationship between the composition of the intestinal microbiota and the level of physical activity. It has been shown that physical training has an impact on the qualitative and quantitative differentiation of microorganisms capable of producing butyrate, e.g., bacteria of the order *Clostridiales* or Firmicutes-types. These changes create a natural barrier that prevents pathogenic bacteria from penetrating the intestinal epithelium (Durk et al. 2018).

O’Donovan et al*.* (2020) analyzed the microbiota of Irish athletes (*n* = 37) from 16 different disciplines. The study groups (disciplines) were divided into those with increased exercise dynamics (based on VO_2_ max) and static (classified according to the maximum isometric contraction). The analyzed samples showed the presence of at least one of the species: *Eubacterium rectale, Polynucleobacter prausnitzii, Bacteroides vulgatus* and *Gordonibacter massiliensis*. Athletes practicing sports with high dynamics in the composition of the microbiota had an increased number of species such as *Lactobacillus acidophilus, Prevotella intermedia* and *Faecalibacterium prausnitzii*, while in the group of people practicing disciplines characterized by both high dynamics and statics, an increased amount of bacteria of the species *Bacteroides caccae* was found. Additionally, in the stool and urine, increased lactate concentration was found in case of static sports and creatinine in case of sports characterized by high dynamics. This is probably due to the increased muscle rotation in dynamic sports, which results in an increase in creatinine levels. These differences may indicate some correlation between the training load and the composition of the intestinal microflora (O’Donovan et al. 2020).

Estaki et al*.* (2016) studies have shown that among physically active people, and the greatest impact on the species variability of microorganisms that make up the intestinal microbiota is achieved by reaching the maximal oxygen uptake also known as maximal aerobic capacity (VO_2_ max), and not by age, sex, BMI or diet as previously thought. In addition, the increased diversity of microorganisms results from their properties such as chemotactic and sporulation abilities or participation in the biosynthesis of fatty acids. In the studies of (Matsumoto et al. 2008) on rats, it was proved that there is a relationship between high cardiorespiratory efficiency and an increase in the amount of short-chain fatty acids (SCFA).

**Fig. 1:**
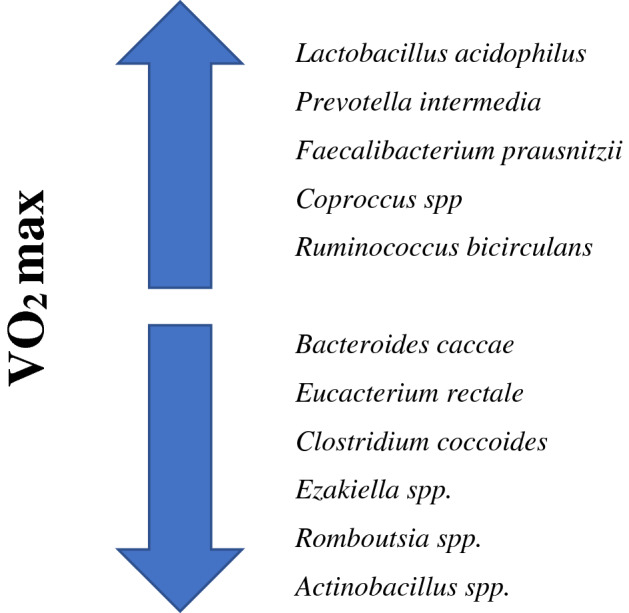
Differences in the composition of microbiota depending on the VO_2_ max

The increased amount of butyrate is associated with the presence of bacteria of the order Clostridiales, families Lachnospiraceae (genus *Roseburia*) and Erysipelotrichaceae. It was also found that there is probably a relationship between VO_2_ max and some properties of microorganisms, e.g., chemotaxis. One possible mechanism may be due to the fact that butyrate, which is abundant in people with higher levels of cardiorespiratory fitness, modulates neutrophil chemotaxis. It is also presumed that the maximum amount of oxygen consumed by the body during intense exercise (VO_2_ max) is inversely correlated with lipopolysaccharide (LPS) biosynthesis. LPS is an element of the cell wall structure of Gram-negative bacteria, which, after entering the bloodstream, exhibits endotoxic properties. Binding of lipopolysaccharide to Toll-like receptor 4 (TLR4) on many types of cells causes inflammation, which is a sign of stimulation of the immune system. Physical activity reduces the amount of LPS and stimulates the immune system. Durk et al*.* (Matsumoto et al. 2008) analyzed the relationship between cardiovascular capacity and the intestinal microflora in women. In this study, it was found that with increasing VO_2_ max, the ratio of the number of bacteria from the Firmicutes family and the genus *Bacteroides* increases. In another study in premenopausal women, those with low VO_2_ showed a reduction in Bacteroides and an increased number of *Eubacterium rectale* and *Clostridium coccoides* (Durk et al. 2018) (Fig. 1).

Very interesting results were obtained during the research on marathon runners. Differences in the composition of the intestinal microbiota before and after the run were found. In the studies by Zhou et al*.* (2014), the differences were shown in bacteria from the Coriobacteriaceae and Succinivibrionaceae families, as well as a reduction in the number of bacteria from the genera *Ezakiella*, *Romboutsia* and *Actinobacillus*, with an increased number of bacteria of the genus *Coprococcus* and *Ruminococcus bicirculans*. (Fig. 1) (Scheimann et al. 2019) conducted research among participants of the Boston marathon. Samples were taken one week before and after the run. Physical activity changed the number of microorganisms constituting the microbiota of the gastrointestinal tract, mainly bacteria of the genus *Veillonella*. These microorganisms convert pyruvate to lactate and acetate via the methylmalonyl-CoA pathway. Increased level of lactic acid, in turn, promotes the colonization of the gastrointestinal tract by bacteria of the genus *Veillonella* (Zhou et al. 2014).

Figure 1 shows changes in the microbiota composition depending on the difference in the VO_2_ max. The species of microorganisms change with the evolution of the ceiling, which means that among endurance athletes, the species will prevail *Lactobacillus acidophilus,* and in people practicing sprint sports *Bacteroides caccae.*

The level of the VO_2_ max achieved, and thus, the composition of the microbiota will be related to various sports disciplines. We will discuss this issue in the next chapter.

## Microbiota and sport disciplines

There are few reports on the relationship between the gut microbiota and various sport disciplines. The research often focuses on people with low physical activity and those who practice amateur sports, i.e., with occasional physical activity. Researchers confirm the relationship between the qualitative and quantitative composition of the gastrointestinal microbiota and physical activity and diet (O’Donovan et al. 2020; Clarke et al. 2014; Petersen et al. 2017).

In a study by Clarke et al*.* (2014) on the microbiota of rugby players, it was found that players with a high body mass index (BMI) had fewer bacteria of the genus *Akkermansia*. This is probably due to the high-protein diet used by these athletes.

Petersen et al*.* (2017) studied the intestinal microbiota of professional and amateur cyclists. They found that the more the contestants train, the greater the proportion of Prevotella bacteria in their microbiota. These microorganisms are involved in the metabolism of carbohydrates and amino acids, including branched-chain amino acids (BCAAs). *Methanobrevibacter smithii* producing methane was more common in professional cyclists. The presence of bacteria of the genus *Akkermansia* was found in most of the tested athletes (Petersen et al. 2017).

In rowers and ultra-marathon runners, *Veillonella* strains were found to be abundant. These microorganisms use lactate as their sole carbon source and are responsible for the conversion of lactate to pyruvate with increasing running time. As a result, it naturally catalyzes the enzymatic process, which results in better sports results (Jang et al. 2019).

Bodybuilders showed a higher percentage of bacteria from the genera *Faecalibacterium*, *Sutterella*, *Clostridium*, *Haemophilus*, and *Eisenbergiella* as compared to professional runners. Much smaller numbers of bacteria belonging to probiotic strains such as *Bifidobacterium adolescentis, Bifidobacterium longum, Lactobacillus sakei* and those producing short-chain fatty acids, such as *Blantia wexlerae* or *Eubacterium hallii*, were also detected. Probably, the selection of such microorganisms is related to the diet used. For bodybuilders, it is most often a diet rich in proteins and fats with a low carbohydrate content, while for athletes, it is a low-sugar, low-fiber diet. This indicates the participation of selected microorganisms in the metabolism of various nutrients (Jang et al. 2019).

Additional literature data on the share of individual types of bacteria depending on the discipline are presented in Table 1.

**Table 1:** Review of the literature data on the share of bacterial species in athletes from various disciplines

Study	Age	Participants (numbers of participants)	BMI	Dietary characteristic	Microbiota
Galle et al.(2020)	18–36	Highly active (*n* = 140)	22.4 ± 2.8	Mediterranean diet	*↓Megasphaera*
					*↓Lachnobacterium*
					*↓Dialister*
					*↓Paraprevotella*
Manor (Han et al. 2020)	49–12	Highly active (*n* = 3400)	27 ± 6	Fruits, vegetables	*↑Ruminococcacee*
					*↑Clostridiales*
					*↑Veillonella*
					*↑Lachnospira*
					*↑Faecalibacterium*
Han (Manor et al. 2020)	12–26	Rowers (*n* = 19)	–	Staple food, vegetables, meat poultry, seafood, bean, grease	*↑Clostridiales*
					*↑Ruminococcaceae*
					*↑Faecalibacterium*
					*↓Bacteroides*
Jang et al. (2019)	20–26	Bodybuilding (*n* = 45)	20.5–28.1 ± 4.2	Diversity in carbohydrate, proteins, dietary fiber, fat	*↑Faecalibacterim*
					*↑Clostridium*
					*↑Eisenbergiella*
					*↑Haemophilus*
					*↓Blautia*
					*↓Leuconostoc*
					*↓Weissella*
					*↓Bacteroides*
					*↓Bifidobacterium*
Scheiman et al. (2019)	–	Runners (*n* = 26)	–	–	*↑Veillonella*
Barton et al. (2018)	–	Rugby players (*n* = 86)	Control group divided for < 25.2 and ≥ 26.5	Proteins, fiber, carbohydrates, sugars, fat	*↑Erysipelotrichia*

In the next chapter, we will discuss the influence of probiotics on the microbiota of the athletes’ digestive tract.

## Influence of a sport diet on microbiome

Achieving the best sports results depends on the proper motor preparation of the body and a properly balanced diet with a specific content of macro- and microelements. For their growth, microorganisms also require the supply of appropriate metabolic substrates and take part in biochemical transformations, which result in various products as SCFA (Mohr et al. 2020; Hughes 2020).

Certain types of microorganisms are directly involved in the metabolism of given dietary components. Bifidobacteria are involved in modulating intestinal homeostasis, local and systemic regulation of the immune response and protect against the development of inflammation. Moreover, these microorganisms belong to the group of acetate-producing bacteria (*Bifidobacterium adolescentis, Blantia wexlerae*). Their abundance in the presence of lactate-producing *Lactobacillus sakei* may lead to a reduction in the number of butyrate-producing Eubacteria (Jang et al. 2019). These bacteria use glycerol to produce reuterin—an antibacterial substance that regulates the metabolism of microorganisms (Jang et al. 2019).

It is very difficult to indicate the place and role of microorganisms in the metabolism of individual nutrients, therefore analyzing these relationships in selected diets is in many cases not justified.

In the diet of athletes, it is recommended to consume a large amount of simple sugars. This is to store the maximum amount of glycogen. As a result, the blood glucose concentration during training is maintained at the correct level. Additionally, a low dietary fiber and starch content may lead to a weakening of intestinal peristalsis, disturbed bowel movements and changes in the diversity of the intestinal flora (Rodriguez et al. 2009). Moreover, it is fiber that influences protein fermentation in the colon and the relative abundance of microorganisms responsible for proteolytic fermentation. Reducing the amount of fiber in the diet reduces the effect of consuming large amounts of protein. Carbohydrates are the preferred carbon source for many gut microbes (Table 2). Protein fermentation occurs when fiber is lacking. Therefore, in case of a high-protein diet, it is recommended to eat large amounts of fiber, which reduce the share of proteolytic fermentation in the metabolism of the colon microflora (Chassard and Lacroix 2013). The source of nutrients reaching the colon is a wide variety of complex glucans, including undigested polysaccharides (cellulose, hemicellulose, lignin, resistant starch pectin and oligosaccharides), but also simple sugars, disaccharides, mucins and mucopolysaccharides. The products of carbohydrate metabolism have a significant impact on the transformation of other nutrients. Some anaerobic-colonizing bacteria use the fermentation of amino acids and carbohydrate metabolites to produce energy (Table 2). Food-derived amino acids or those released from endogenous sources and reaching the colon can serve as a source of amino acids for protein fermenters (Karlund and Gomez-Gallego C. et al. 2019). Microbial fermentation of amino acids and carbohydrates in the large intestine leads to the formation of SCFA precursors. Acetate, butyrate and pyruvate are formed. All of them have a positive effect on the human body. Butyrate is used as an energy source by the colonocytes, while pyruvate is involved in hormonal regulation. Acetate affects ex. on reducing of hypertension (Diether and Willing 2019). The fermentation of amino acids also produces branched-chain fatty acids such as methyl valerate, isovalerate, isobutyrate and α-butyrate. They are recognized as reliable biomarkers of proteolytic fermentation. Diets rich in proteins primarily contribute to building muscle mass in the body (Table 2). However, excessive consumption of high-protein products may lead to the accumulation of excess nitrogen substrates for putrefactive bacteria, the products of which are metabolized: ammonia, hydrogen sulfide, amines, thiols and indoles. (Table 2) As the food content moves, the carbohydrate content decreases, and the protein fermentation products become more and more harmful. Excessive protein consumption can damage the DNA in the cells of the colon mucosa (Davila et al. 2013; Ríos-Covián et al. 2016; Bourne 2016).

**Table 2:** Microorganisms involved in the metabolism of nutrients

Nutrient		Genera of microorganisms
Dietary fiber	Oligosaccharides	*Bacteroides*
		*Prevotella*
		*Ruminococcus*
		*Roseburia*
		*Faecalibacterium*
		*Bifidobacterium*
Lactose		*Lactobacillus* *Bifidobacterium*
Mucins	Mucopolysaccharides	*Akkermansia* *Bacteroides*
Proteins		*Clostridium* *Desulfovibrio* *Peptostreptococcus* *Acidaminococcus* *Veillonella*

## The influence of probiotics on the sportsman microbiome

The pioneer nations in using researchers in sport were the Soviet Union and former Eastern Bloc countries. What started with the space race with cosmonauts then moved onto athletes, who were closely scrutinized and analyzed by medical doctors, physiologists, microbiologists, dietitians and other specialists using a very different approach than their Western counterparts, where trainers placed most effort on the athletes physical training (Xue et al. 2017). Beneficial bacteria in the diets of sportsmen were a common practice back then since 1950s, long before the era of doping dominated worldwide sports.

Probiotic bacteria are live bacterial strains that, when administered in appropriate amounts, benefit the host organism. Strains belonging to the genera: *Bifidobacterium, Lactobacillus* and other lactic acid bacteria—*Lactococcus, Streptococcus* and fungi of the genus *Saccharomyces* are most often used as probiotic bacteria. Probiotic strains may influence the changes in the population of microorganisms constituting the intestinal flora and control the functioning of this ecosystem (Bagarolli et al. 2017). The human gut is a system in which nutrients, microbiota, and host cells interact in various ways. The relationship between microorganisms and host cells can be both negative (pathogenicity, toxigenicity, translocation of microorganisms) and positive. Many studies show that regular consumption of probiotics can positively affect changes in the population and structure of the gut microflora. As a result, the functionality of the immune system changes and the proliferation of intestinal epithelial cells increases, which is often observed in people actively practicing sports (Mackos et al. 2013). The combination of prebiotics, or fermented nutrients, including fructans and oligosaccharides, and foods enriched with *Lactobacillus* and *Bifidobacterium* strains can alter bowel function. The administration of probiotics containing *Lactobacillus rhamnosus* strains to patients had an effect on maintaining the proper functioning of lymphoid tissue, modulating the immune system response and lipid metabolism in the intestinal epithelial tissues (Mackos et al. 2013; Jacouton et al. 2015).

Research shows that supplementing the diet of athletes with probiotics is of great importance. Physical activity and diet contribute to a greater variation in the composition of the intestinal flora in athletes compared to people who lead a sedentary lifestyle. During maximum exertion, visceral blood flow can be reduced by up to 80% to ensure sufficient blood flow to actively working muscles. Research also confirms the cascade of exercise intestinal permeability, which results in an increased risk of bacterial translocation after exercise (Chantler et al. 2021). March et al*.* (2017) demonstrated a significant correlation between post-exercise intestinal injury and the ratio of plasma bacteria to total bacterial DNA, reflecting increased bacterial translocation. Other researchers also show an increase in inflammatory markers after exercise (Snipe et al. 2018; Zuhl et al. 2014; Lamprecht et al. 2012). The consumption of probiotics may help to reduce inflammation. Supplementation with probiotics or prebiotics stimulates the multiplication of bacteria contained in them (*Bifidobacterium, Lactobacillus*) and may improve metabolism and increase immunity. The use of probiotic preparations reduces the level of zonulin—a modulator of intestinal permeability (Fasano 2011).

Consumption of probiotic preparations containing the following strains: *Bifidobacterium bifidum, Bifidobacterium lactis, Lactobacillus acidophilus, Lactobacillus brevis,* and *Lactococcus lactis* helps to maintain the redox balance and reduces the susceptibility to inflammation in people undergoing physical exercise. Although the mechanism of this interaction has not been elucidated, it is probably due to a direct interaction between the intestinal microbiota, the immune system within the mucous membranes, and the modulation of the functions of pulmonary macrophages and T lymphocytes. The results of studies carried out on long-distance runners indicate a better adaptation of their respiratory system to physical exertion after using probiotics (West et al. 2009). This is due to the effect on IFN-α, which stimulates the enzyme involved in the catabolism of tryptophan—indoleamine 2,3-dioxygenase (Strasser et al. 2016).

There are little data on the effect of probiotics on the gut microbiota in athletes. Supplementing the diet of athletes with various species of probiotic bacteria is probably necessary to maintain the proper functioning of the gut–brain axis. The psychological aspect of the sport has been the subject of many studies up to date. It remains clear that to become a winner, the athlete must not only have the required physical but also very good mental preparation. The influence of gut microbiota on psychological well-being is another aspect that requires much broader studies in sports. The gut microflora influences many aspects of human biology. Establishing a diet containing probiotic bacteria for athletes should be the subject of further research.

Bacteriophages, viruses specifically infect and kill bacteria, could be an example of a dark matter in the human microbiome. Phages, for short, are one of the most numerous entities on the earth (Wojciuk et al. 2019). Bacteriophage-related research focuses mainly on their therapeutic use, and there is a huge gap in knowledge on the influence of phages on organisms’ performance (Grygorcewicz et al. 2020, 2021; Sutton and Hill 2019). This should be noted that phages as specific bacterial predators infect susceptible cells and influence on bacterial community in the human gut (Sausset et al. 2020). The reduction of specific bacterial species may promote the growth of others which may influence directly the microbiome composition. The search for the relation between phageome composition and sports needs future research.

## Conclusions

Modern methods of molecular biology are a very good diagnostic tool that allows to learn about the mechanisms at the level of microorganisms that directly affect the functioning of the human body. Most of the literature data indicate a significant share of *Clostridiales* bacteria, such as *Bacteroides, Lactobacillus, Bifidobacterium,* or *Veillonella*. However, there is still no clear answer, which is the dominant species colonizing the gastrointestinal tract of athletes. The diversification of the intestinal microbiota also changes depending on the consumed diet. A high-fiber diet increases the number of bacteria from the genera *Lactobacillus* and *Bifidobacterium*, but proteins are the most crucial building blocks of muscle mass. They are involved in their metabolism, among other bacteria of the genus *Clostridium* and *Veillonella*, in which the increase was observed in marathon runners. Despite the reports of many authors on the microbiota of the gastrointestinal tract in athletes, it is still difficult to unambiguously link the composition of the bacterial flora and sport discipline with diet. Determining the correlation between them can be an important indicator that will allow sportsmen and women to achieve better and better results.
